# Exploring amino acid and peptide transporters as therapeutic targets to attenuate virulence and antibiotic resistance in *Staphylococcus aureus*

**DOI:** 10.1371/journal.ppat.1009093

**Published:** 2021-01-14

**Authors:** Merve Suzan Zeden, Órla Burke, Moya Vallely, Claire Fingleton, James P. O’Gara

**Affiliations:** Microbiology, School of Natural Sciences, National University of Ireland, Galway, Ireland; Tufts Univ School of Medicine, UNITED STATES

*Staphylococcus aureus* continues to pose a major threat to public health and is responsible for 20,000 deaths each year in the US [1]. The problem is exacerbated by methicillin-resistant *S*. *aureus* (MRSA) which, according to the World Health Organisation’s 2014 Antimicrobial Resistance Global Report on Surveillance is associated with a >60% increase in mortality compared to antibiotic susceptible *S*. *aureus*. Membrane transport systems can control both virulence and antibiotic resistance and represent novel targets for therapeutic agents. Here we discuss how efforts to overcome antimicrobial drug resistance could include novel agents targeting important metabolic processes dependent on membrane transporters, which have the potential to augment existing antibiotics.

## Why investigate amino acid and peptide transporters as potential therapeutic targets?

Amino acids are essential for sustaining cell integrity and metabolic homeostasis. In addition to protein synthesis, amino acids are also precursors for biosynthesis of nucleotides, lipids and cell wall components. *S*. *aureus* can synthesize many of these amino acids but will often preferentially transport them into the cell from the external environment [2].

Limited glucose availability (for example in an abscess) represents an environment in which catabolism of peptides or amino acids is important for *S. aureus* growth [3]. Bioinformatic analysis reveals several pathways that enable *S*. *aureus* to catabolize multiple amino acids, which in turn can generate key central metabolic intermediates such as pyruvate, oxaloacetate and 2-oxoglutarate. Reflecting this importance of amino acids in metabolism, *S*. *aureus* has multiple oligopeptide permeases, free amino acid transporters and proteases to degrade host proteins.

An analysis of 64 *S*. *aureus* strains revealed that amino acid metabolism genes are disproportionately associated with the pangenome [4] indicating that targeting transporters associated with core amino acid metabolism is likely to have broader therapeutic potential against diverse *S*. *aureus* isolates. The diversity and redundancy of amino acid, peptide, osmolyte and nucleoside uptake systems also presents a significant challenge. There are at least 292 genes in the USA300_FPR3757 genome predicted to encode membrane transporters, of which 120 appear to be associated with amino acid, osmolyte or nucleoside transport.

Bioinformatic tools are generally helpful in identifying and predicting the functions of putative transporters, but experimental work is required to verify the substrates transported by permeases and their physiological roles. Historically, studies on bacterial membrane transport systems focused on their contribution to growth and physiology *in vitro*. However, new data on nutrient availability and metabolism in niche-specific models of *S*. *aureus* infection suggests that amino acid transporters may also contribute to virulence and antibiotic resistance [5–19]. In this context, renewed effort to understand how, why and when amino acids are taken up by *S*. *aureus* cells under controlled laboratory conditions, and *in vivo*, may identify specific membrane transporters as novel and potentially druggable therapeutic targets. Such studies need to focus on the role of these transport systems in metabolism, as well as their contribution to virulence, and resistance to existing antimicrobial drugs. Analogues of substrates for membrane transporters implicated in virulence and/or drug resistance may provide a starting point for novel antimicrobial agents, which are less likely to impose a selective pressure for the emergence of resistance. Future clinical trials would be needed to investigate the relevance of this therapeutic approach in humans or animals.

### What effect does the environment have on amino acid transport, and what does this mean for virulence and resistance?

*S*. *aureus* can alter the total intracellular amino acid concentration and relative abundance of individual amino acids in response to external osmotic and pH stress [2]. Mild ethanol stress was shown to be associated with impaired acetate catabolism and ammonia accumulation and directed the specific uptake of individual amino acids from the culture medium [20]. This selective transport of amino acids may be driven by specific requirements for stress-responsive protein synthesis, and/or stress-driven redirection of metabolic activity [21]. Nutrient limitation and oxygen availability also impact amino acid uptake and catabolism, including during planktonic and biofilm growth [22,23].

Intracellular amino acid concentrations influence physiology and virulence levels by controlling the activity of the stringent response (ppGpp) and c-di-AMP nucleotide signaling systems, and the global transcriptional regulator CodY. Amino acid starvation-mediated activation of the stringent response and increased c-di-AMP levels have pleiotropic effects including expression of high-level β-lactam resistance [24–26]. When GTP and branched-chain amino acid (BCAA) levels are reduced under nutrient-limiting conditions, genes normally repressed by CodY are activated and collectively play a role in adaption to starvation. CodY also regulates several virulence factors [5,7], highlighting a link between environmental conditions, metabolism, and virulence.

### Do all amino acid transporters have the same effects on growth, virulence and resistance?

Transport of several amino acids has been shown to be important for *S*. *aureus in vivo* survival, virulence and drug resistance. Interplay between these transporters in different growth media or host niches reveals both the complexity of amino acid transport mechanisms, as well as possible therapeutic opportunities to manipulate their activity in order to affect changes in virulence and/or antibiotic resistance.

#### BcaP and BrnQ

*S*. *aureus* transports BCAAs using BcaP and BrnQ1-2-3 [5,7,8]. *In vitro*, BrnQ1 is the primary transporter of leucine and valine, whereas BcaP plays a more significant role *in vivo*. Interestingly, a *brnQ2* mutant was hypervirulent in a systemic infection model, potentially due to impaired isoleucine transport causing de-repression of CodY-controlled virulence genes, highlighting the importance of experimentally verifying the suitability of membrane transporters as drug targets.

#### PheP

Mutation of predicted phenylalanine permease gene *pheP* impacted growth [19] and attenuated virulence [18]. The lysine analogue S-(β-aminoethyl)-L-cysteine (thiosine) can inhibit lysine transport by the *E*. *coli* PheP homologue, LysP, suggesting that inhibition of PheP with a phenylalanine analogue may attenuate *S*. *aureus* virulence.

#### PutP

Mutation of high-affinity proline transporter PutP, which is up-regulated by nutrient depletion [14], attenuates virulence in a rabbit endocarditis model [13] even though OpuD remains functional as a low-affinity proline transporter [15], suggesting that drug-mediated inhibition of PutP-mediated proline transport may have therapeutic potential.

#### GltT

The major aspartate transporter GltT is important for *S*. *aureus* survival in a mouse model of osteomyelitis. Although GltT does not transport glutamate [16], high concentrations of glutamate in bone tissue block GltT activity including GltT-mediated aspartate transport, making *de novo* aspartate biosynthesis essential for *S*. *aureus* survival and persistence in this niche [16]. A *gltT* mutant exhibited increased sensitivity to heat shock, acetic acid and gentamicin stress [17].

#### GlnPQ and AlsT

Mutation of the predicted glutamine transporter GlnPQ increased TCA cycle activity, decreased polysaccharide intercellular adhesin biosynthesis, and significantly reduced virulence in a rabbit endocarditis model [12]. Recently it was demonstrated that GlnPQ does not transport glutamine and that AlsT is instead the main glutamine transporter [9]. Moreover, AlsT-mediated glutamine uptake decreased c-di-AMP levels, which is known to effect cell envelope homeostasis, virulence and β-lactam resistance [9,25].

#### CycA

Impaired transport of alanine in a *cycA* mutant increased MRSA susceptibility to oxacillin and d-cycloserine (DCS), an alanine analogue antibiotic that interferes with peptidoglycan (PG) biosynthesis [10]. Mutation of *cycA* mutant or exposure to DCS had similar effects on PG structure [10], revealing interplay between alanine transport and susceptibility to β-lactam antibiotics. β-lactam/DCS combinations acted synergistically against MRSA in a mouse bacteremia model [10], suggesting that drug-mediated interference with CycA might ameliorate β-lactam resistance in MRSA.

#### TycP and TycABC

The low-affinity transporter TcyABC and the high-affinity permease TcyP, which transport the sulfur-containing amino acids cysteine and cystine, were recently implicated in virulence using a mouse model of systemic infection [11]. However, a double TcyP-TcyABC mutant still established infection, indicating that an alternative sulfur transporter(s) may facilitate glutathione transport, potentially complicating putative strategies to target sulfur transport using cysteine/cystine analogues.

### How can amino acid and peptide transport activity be exploited to develop new therapeutic approaches for *S*. *aureus* infections?

Amino acid transporters are among the most abundant membrane proteins in *S*. *aureus*, with niche- and environment-specific roles in maintaining cell integrity and metabolic homeostasis in infected host tissue. Further advances in our understanding of the mechanisms underpinning the contribution of amino acid permeases to virulence and antibiotic resistance may identify new drug targets for which the natural substrates can be identified. The therapeutic potential of amino acid and peptide analogues has been explored for diseases ranging from diabetes to cancer. For example the glycine analogues, glyphosate and aminomethylphosphonic acid successfully inhibited growth in eight human cancer cell lines, but not two immortalized human normal prostatic epithelial cell lines [27].

An attractive feature of this strategy is the possibility of using amino acid analogues or new drugs derived from amino acid analogues as lead compounds in studies to evaluate the physiological impact of interfering with amino acid transport systems on the metabolism, growth and virulence of *S*. *aureus*. The activity of amino acid analogue antibiotics such as D-cycloserine and β-chloro-D-alanine against *S*. *aureus* demonstrates the effectiveness of drugs based on amino acid analogues [10,28]. Combining new drugs targeting amino acid transporters may also enable re-purposing of other antibiotics as part of efforts to overcome resistance in *S*. *aureus* and MRSA. Nevertheless, translation of this anti-transporter approach into clinical practice will encounter several obstacles. Multiple substrates for some transporters may complicate this approach. In addition, drugs identified *in vitro* may have limited activity *in vivo* or unwanted activity against host cell membrane transporters and the beneficial microbiota. Strategies to mitigate potential off-target side effects in a clinical setting will be needed to realize the therapeutic potential of this approach.
